# Cellulose nanofibers/polyvinyl alcohol blends as an efficient coating to improve the hydrophobic and oleophobic properties of paper

**DOI:** 10.1038/s41598-022-20499-8

**Published:** 2022-09-27

**Authors:** Shancong Huang, Xiyun Wang, Yu Zhang, Yu Meng, Feiguo Hua, Xinxing Xia

**Affiliations:** 1 College of Textile Science and Engineering (International Institute of Silk), Zhejiang Sci-Tech University, Hangzhou, 310018 Zhejiang China; 2 Zhejiang Jinchang Specialty Paper Co., Ltd., Quzhou, 324404 Zhejiang China

**Keywords:** Nanoscale materials, Structural materials

## Abstract

The effect of cellulose nanofibers (CNFs)/polyvinyl alcohol (PVA) coating on the hydrophobic, oleophobic, and strength properties of paper were investigated. The results showed that the size of bamboo fibers (BFs) decreased significantly and the crystallinity increased significantly after biological enzyme treatment. The average length of CNFs obtained by high pressure homogenization was 2.4 µm, the diameter was 28.7 nm, and the crystallinity was 63.63%. When the coating weight of PVA/CNF was 2.0 g/m^2^ and the CNF dosage was increased from 0.0% to 3.0%, the paper grease resistance grade was increased from 7 to 9, the Cobb value was decreased from 22.68 ± 0.29 g/m^2^ to 18.37 ± 0.63 g/m^2^, the contact angle was increased from 67.82° to 93.56°, and the longitudinal and transverse tensile index were increased from 67.72 ± 0.21 N m/g and 37.63 ± 0.25 N m/g to 68.61 ± 0.55 N m/g and 40.71 ± 0.78 N m/g, respectively. When the CNF dosage was 3.0% and the coating weight of PVA/CNF was 4.0 g/m^2^, the grease resistance grade of the paper was 12, the Cobb value was 21.80 ± 0.39 g/m^2^, and the longitudinal and transverse tensile indices were 72.11 ± 0.43 N m/g and 42.58 ± 0.48 N m/g, respectively. In summary, the increase of CNFs can effectively improve the lipophobicity, hydrophobicity and tensile strength of the PVA coated paper.

## Introduction

Grease-proof paper is a kind of paper with resistance to grease penetration and absorption, which is used in industrial, medical, food, and other packaging fields^1^. Most paper cannot prevent the decay and deterioration of goods by preventing grease, water, and oxygen transmission^2^. Therefore, plastic films such as polyethylene (PE) and polypropylene (PP) are used to improve the hydrophobic and oleophobic properties of paper^3^. However, most petroleum-based plastics are non-biodegradable, non-renewable, and have low recyclability^4^. Moreover, microplastics also threaten human health through the food chain^5^. In addition, fluorine-containing oil repellent is widely used in the production of grease-proof paper as an excellent grease-proof coating liquid^6^. But most fluorinated packaging materials release non-biodegradable perfluoroalkyl substances during the composting process, which are not only harmful to the environment but also to human beings^7^. Thus, safe, and environmentally friendly new fluorine-free oil-repellent materials have become a research hotspot, such as degradable polymers, nanocellulose, and chitosan, et al.^8–10^.

The porous structure of paper is inherently oil-absorbing by means of capillary force, and cellulose surface is also oleophilic, therefore, surface coating is an effective method to prevent oil/grease from penetrating into the interior of the paper according to the pore blocking theory^11^. Polyvinyl alcohol (PVA) is a white water-soluble polymer compound with strong adhesion, smoothness, oil resistance, and solvent resistance^12–14^. It is an environmentally friendly functional material that can be degraded into water and carbon dioxide by bacteria and enzymes^15^. Therefore, PVA is widely used in papermaking as an environmentally friendly oil repellent material. The PVA coating can significantly improves the oleophobicity of paper, but the hydrophobicity of paper decreases due to the hydrophilicity of PVA. Shen et al.^16^ reported that the addition of alkyl ketene dimer (AKD) in PVA coating improved the hydrophobic property of paper, but the grease-proof property decreased. Therefore, the hydrophilic property of PVA limits its application as an oil repellent material.

Nanocellulose is a cellulose particle with a size of less than 100 nm in one dimension, which is obtained by the chemical or mechanical processing of plant fibers. Research shows that nanocellulose products have great strength and function^17^. To realize the special application of nanocellulose, they are divided into various types, including microcrystalline cellulose (MCC), microfibrillated cellulose (MFC), cellulose nanofibers (CNFs), cellulose nanocrystals (CNCs), nanorods, and cellulose whiskers, etc.^18–21^. Compared with plant fibers, CNFs have greater advantages in aspect ratio and specific surface area, with a diameter of 2–100 nm and a length of 500–1000 nm^22^. Turbak et al.^23^ and Herrick et al.^24^ first used a high-pressure homogenizer to treat wood pulp to prepare CNFs with a diameter of less than 100 nm in 1983. The raw materials for preparing CNFs are not only wood, but also seed fibers, bast fibers, grasses, etc. The properties of CNFs prepared from different raw materials are also different. In order to obtain CNFs with a purer concentration, bamboo pulp fibers with less lignin content were selected as raw materials in this paper^25^.

Furthermore, Belbekhouche et al.^26^ demonstrated that CNF films are more beneficial than CNC films for barrier properties. Because the surface of CNFs contains a lot of hydroxyl groups, the CNF films have better barrier properties^27^. Wang et al.^28^ demonstrated that the CNF coating provided excellent hydrophobic properties to the paper. To further improve the hydrophobic properties of CNF coated paper, Mertaniemi et al.^29^ sprayed CNFs on the glass surface, and then used tridecafluoro-1,1,2,2-tetrahydrooctyl)trichlorosilane (FOTS) to modify it by fluorination. Studies have shown that CNF films also have certain oil/grease resistance^30^. Koppolu et al.^31^ used nanocellulose and polylactic acid (PLA) as coating raw materials to prepare barrier packaging paper with both hydrophobicity and oleophobicity. Therefore, the development of CNFs has offered a new alternative to the polymer coating to form a barrier layer^32,33^.

Generally, CNFs were used as a coating to improve air resistance, oil resistance, surface strength, stiffness and tensile strength of paper, while Cobb index and roughness decreased^34,35^. In addition, there are several researchers about the use in combination of PVA and CNFs. Chaabouni et al.^36^ added CNFs to PVA to prepare adhesives and found that the viscosity and water resistance of the adhesives increased, and strongly enhanced the mechanical performance in wet conditions. Deng et al.^37^ prepared CNFs-PVA composite films and found that there are strong interaction and good compatibility between CNFs and PVA molecules. Furthermore, the light transmittance and thermal expansion coefficient of the composite films were decreased, while the tensile strength, Young's modulus, glass transition temperature, and thermal stability were all improved compared with the PVA films^38^. CNFs and PVA are used in combination in some fields such as films, adhesives, and aerogels^39,40^. However, no researchers have studied the hydrophobic and oleophobic properties of PVA/CNF as coating.

In this study, we investigated the properties of CNFs and the effect of CNFs on the hydrophobicity, oleophobicity, and strength of PVA coated paper. CNFs were prepared from bamboo pulp fibers and mixed with PVA to prepare PVA/CNF coating solution. The hydrophobic and oleophobic PVA/CNF coated paper was obtained by a coating method, which was also degradable, recyclable, and environmentally friendly.

## Materials and methods

### Materials

Bleached bamboo pulp (beating degree 13.5ºSR, wet weight 15.13 g) and base paper (110 g/m^2^) were obtained from Zhejiang Jinchang Specialty Paper Co., Ltd. (Quzhou, China). The bleached bamboo pulp composition: cellulose (94.37 wt%), hemicellulose (4.74 wt%), lignin (0.67 wt%), ash (0.10 wt%), and extractive (0.12 wt%). Bleached bamboo pulp Cellulase (enzyme activity 16,000 HCU/g) was obtained from Zhejiang Jinjiahao Green Nanomaterials Ltd. (Quzhou, China). Polyvinyl alcohol (analytical reagent (AR) grade), citric acid (AR grade), sodium citrate (AR grade), and other chemical reagents were purchased from Sinopharm Chemical Reagent Co. Ltd. (Shanghai, China).

### Preparation of cellulose nanofibers

Bamboo pulp was dispersed in water, then 60 U/g of cellulase and citric acid-sodium citrate buffer solution were added, and the reaction was carried out at pH 5.0 and temperature of 50 °C for 6 h, and the enzyme reaction was terminated at 90 °C. Then, the dispersed bamboo pulp was centrifuged at 8000 rpm for 15 min, and the undissolved part was precipitated, which was the enzyme-treatment bamboo fibers (EBFs). The EBFs were homogenized by a high-pressure homogenizer (AH-pilot2018, Shanghai Dibosi Biotechnology Co., Ltd., China) to obtain CNFs.

### Preparation of CNF/PVA composite coating

PVA and CNFs were fully dissolved and mixed in 90 °C water, and then dispersed in an ultrasonic cleaner (KQ-30DE, Kunshan Ultrasonic Instruments Co., Ltd., China), then the uniform CNF/PVA composite coating was obtained.

### Preparation of CNF/PVA coated paper

**Figure 1:**
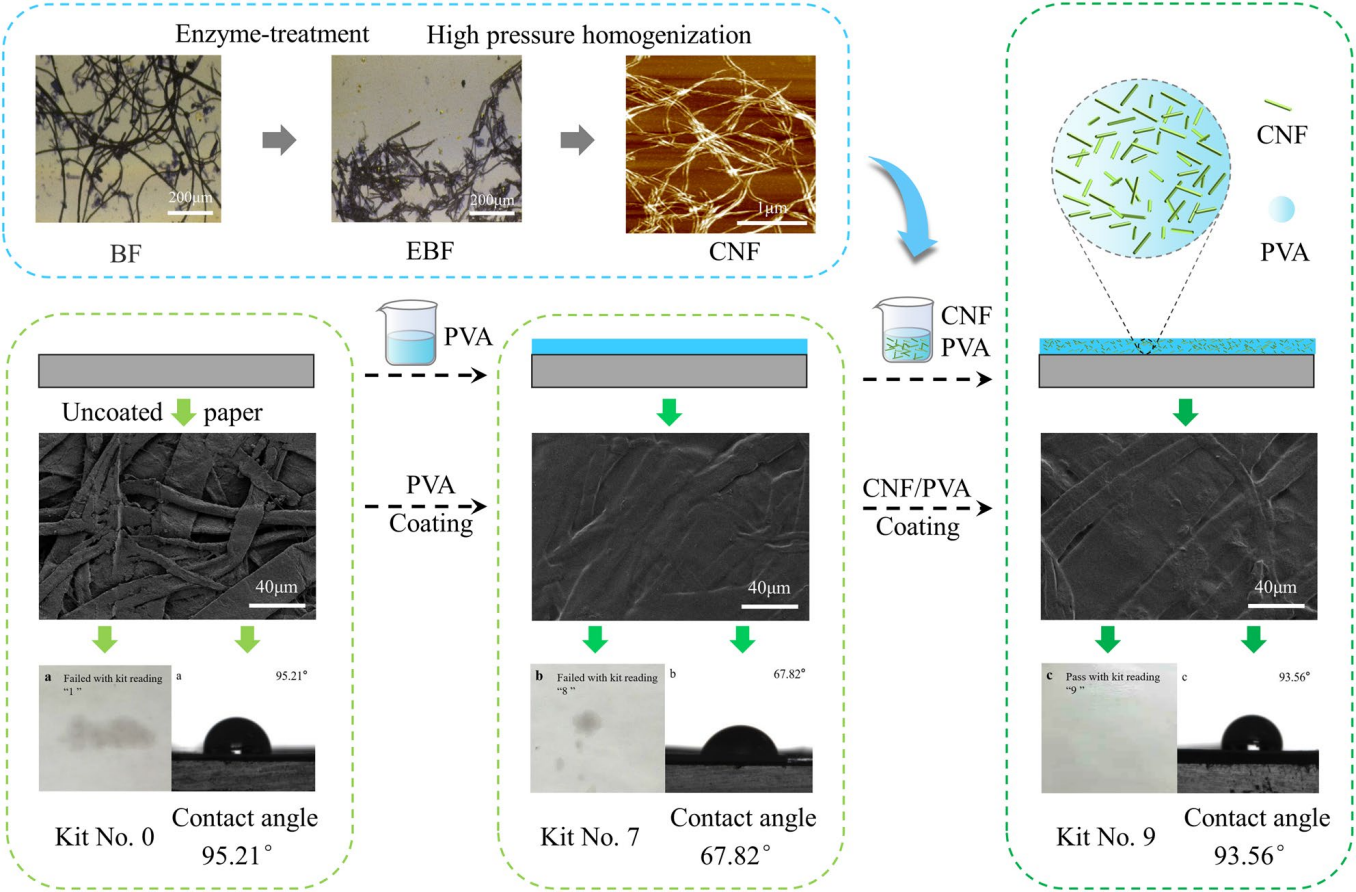
The preparation process and properties of PVA/CNF coated paper.

The prepared composite coating was coated on the surface of the base paper by a manual coater (ZAA 2300, Zehntner, Switzerland), and then dried at 80 °C to obtain the paper with hydrophobic and oleophobic properties. The preparation process and properties of PVA/CNF coated paper are shown in Fig. 1.

### Characterization

The morphology of bamboo fibers (BFs) and EBFs were detected by fiber analyzer (LDAO2, OpTest, Canada) and optical microscope (S800T-930HD, Jiangxi Beitekar Photoelectric Technology Co., Ltd., China). The surface morphology of CNFs was observed by atomic force microscope (XE-100E, PSIA, Korea). The atomic force microscope images of CNFs were imported into Nanoscope analysis and Nano measurer software, and 50 CNFs were randomly selected to measure their length and diameter. In addition, the obtained data were graphed and analyzed using Oringin software. The length and diameter of CNFs were analyzed using Nanoscope analysis software. The microstructure of BFs, EBFs, and CNFs was analyzed by infrared spectrometer (Nicolet 5700, Thermo Fisher Scientific, USA) and X-ray diffractometer (ARL XTRA, Thermo ARL, Switzerland). The samples were scanned at 40 kV and 30 mA in a 2 h range between 10° and 40° using Cu Kα radiation (λ = 15.4 × 10^–2^ nm) at 1°/min^41^. The crystallinity index (CrI) was calculated according to the empirical method developed by Segal (1959) using the Eq. (1) ^42^.

1 $$CrI\left(\%\right)=\frac{{I}_{200}-{I}_{am}}{{I}_{200}}$$

where I_200_ is the maximum intensity of the (200) plane (I_200_, 2θ = 22°) that represents the crystalline and amorphous material, and I_am_ is the minimum intensity between plane (110) and (200) (I_am_, 2θ = 18°) that represents the amorphous material.

The viscosity and pH of the coatings were measured with a viscometer (ROTAVISC lo-vi Complete, IKA, Germany) and a pH meter (PHS-3E, Shanghai Yidian Scientific Instruments Co., Ltd., China). The oil/grease resistance of paper was measured using the TAPPI T559 standard. The contact angle of paper was measured according to ASTM D 724–1999 standard, using a surface contact angle tester (OCA40, Dataphysics, Germany). Water absorption of paper (Cobb test) was determined using Paper Cobb Water Absorption Tester (PWA-01, Sichuan Changjiang Paper Instrument Co., Ltd., China) according to TAPPI T441 standard. The tensile strength of paper was determined using a computerized tensile tester (TTM, Hangzhou Qingtong Boke Automation Technology Co., Ltd., China) according to the TAPPI T494 standard. The surface morphology of the paper was observed using a scanning electron microscope (Phenom pro, Phenom Scientific Instruments (Shanghai) Co., Ltd., China).

## Results and discussion

### Characterization of fiber

#### Morphology analysis

**Figure 2:**
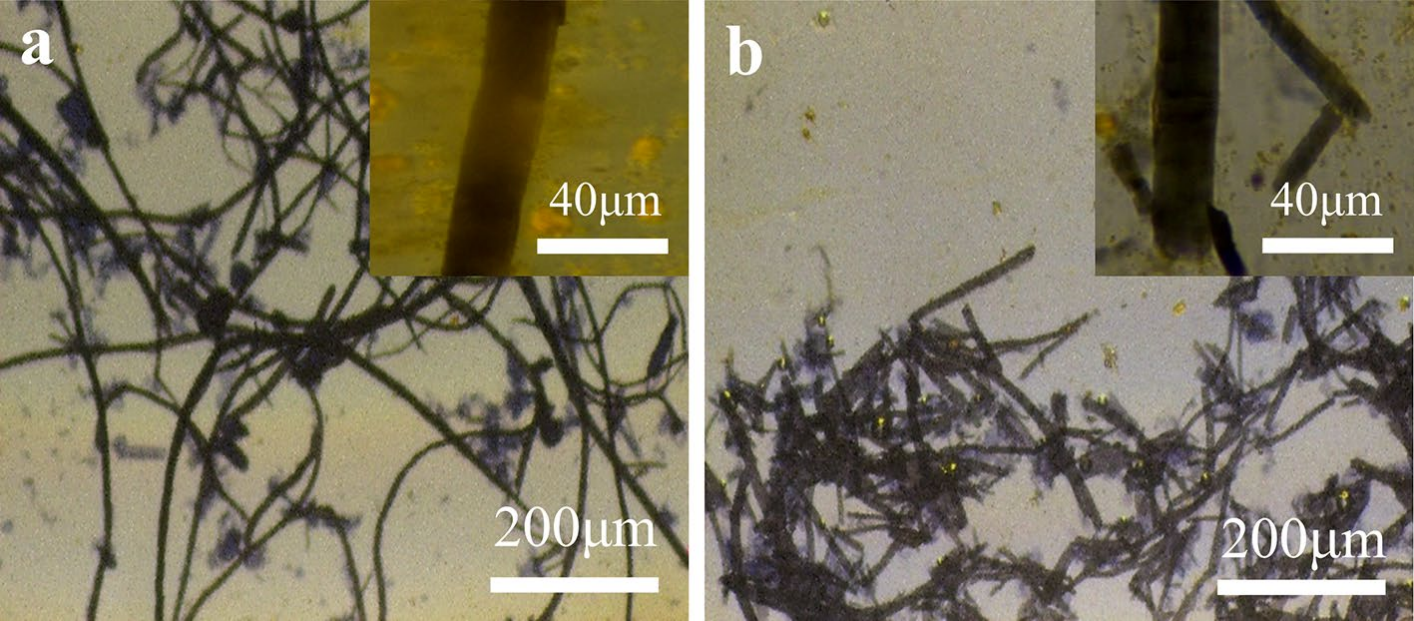
Optical microscope images of (**a**) bamboo fibers and (**b**) enzyme-treatment bamboo fibers.

Figure 2 shows the optical microscope images of BFs and EBFs. Compared with BFs, the diameter of EBFs did no change significantly, about 20 µm. The average length of the EBFs was 0.4 mm, which was 1.1 mm smaller than the diameter of the BFs (Table 1). The reason for this phenomenon was that the amorphous region of cellulose was hydrolyzed by cellulase^43^. The reduction of fiber length was beneficial to the exfoliation of microfibrils from the fibers during the preparation of nanocellulose.

**Table 1:** Physical properties of BFs and EBFs.

Type	Fiber length (mm)		Width (µm)	Coarseness (mg/m)	Curl Index (%)	Fine fiber content (%)	
	Length weighted	Arithmetic mean				Length	Area
BFs	1.534	1.021	23.6	0.2175	16.8	66.9	27.21
EBFs	0.405	0.334	20.3	0.4296	5.9	90.3	74.40

**Figure 3:**
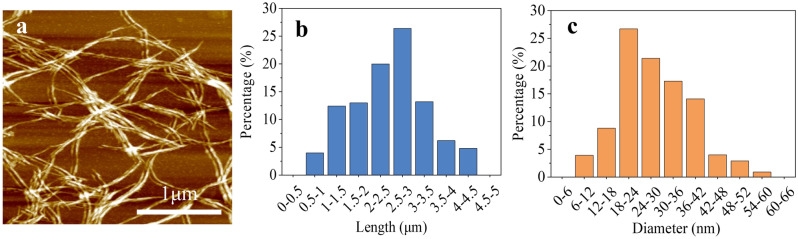
Atomic force microscope images of (**a**) CNFs; (**b**) CNF length and (**c**) CNF diameter.

Figure 3a shows the image of CNF tested with the atomic force microscope (AFM). As shown in Fig. 3a, the CNF was a filamentous structure. And the length and diameter of CNFs were mostly distributed between 1–3.5 µm and 18–42 nm, with an average length of about 2.4 µm and an average diameter of about 28.7 nm (Fig. 3b,c).

#### FTIR spectroscopy analysis

**Figure 4:**
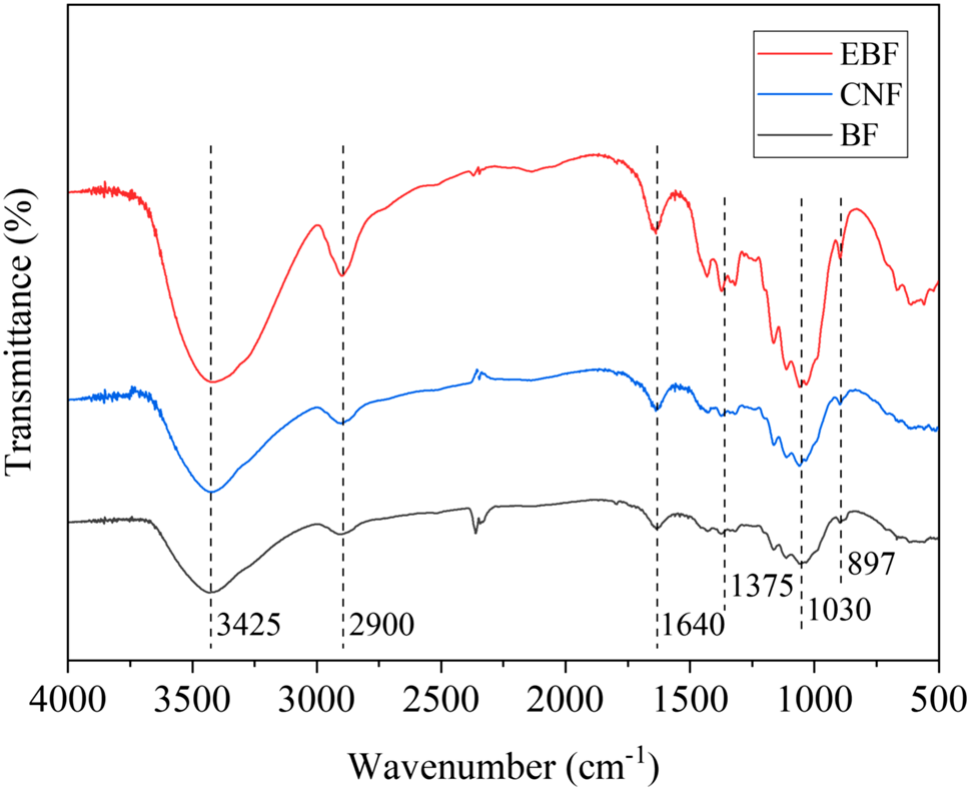
FTIR spectra of BFs, EBFs, and CNFs.

Figure 4 shows the Fourier transform infrared (FTIR) spectra of BFs, EBFs, and CNFs. It could be seen from the figure that EBFs, CNFs, and BFs had similar characteristic peaks. They all contained the characteristic peaks representing cellulose, such as the stretching vibration peak of –OH at 3425 cm^−1^, the stretching vibration peak of –CH at 2900 cm^−1^, and the bending vibration peak of –CH at 1375 cm^−1^^44^.

Compared with BFs, the absorption peaks of EBFs and CNFs were enhanced. The stretching vibration peak of –CO at 1030 cm^−1^ was enhanced, indicating that some of the cellulose of EBFs was hydrolyzed, and the content of primary hydroxyl groups increased^45^. The enhancement of the peak at 897 cm^−1^ was due to the breakage of some β-glycosidic bonds and the destruction of cellulose molecular chain^46^. In summary, EBFs and CNFs still maintained the basic chemical structure of cellulose.

#### X-ray diffraction analysis

**Figure 5:**
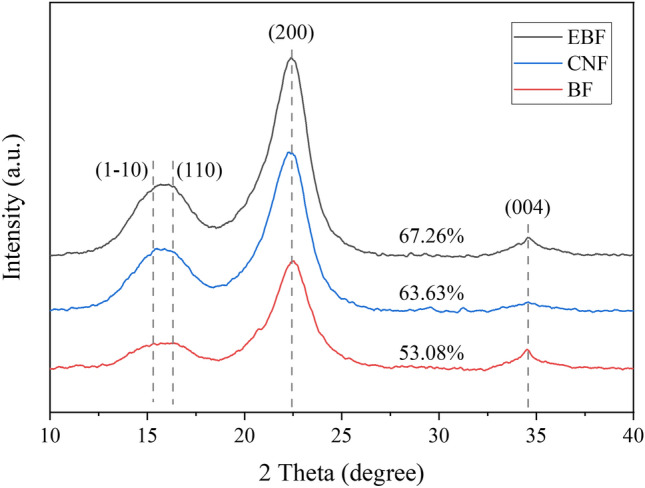
XRD patterns of BFs, EBFs, and CNFs.

Figure 5 shows the X-ray diffraction (XRD) patterns of BFs, EBFs, and CNFs. The diffraction overlapped peak at about 16° correspond to the (1–10)/(110) cellulose crystallographic plane, and the diffraction peaks of 22° and 34.5° correspond to the (200) and (004) cellulose crystallographic plane, respectively, which belong to the typical cellulose type I crystal^47,48^. Compared with BFs, the crystallinity of EBFs increased from 53.08% to 67.26%, which was due to the amorphous region of cellulose being destroyed by enzymes^49^. Compared with BEFs, the crystallinity of CNFs decreased slightly to 63.63%, which indicated that high-pressure homogenization has a certain destructive effect on the crystalline region of cellulose.

### Base paper and coating analysis

Table 2 shows the physical properties of the base paper. The Kit number of paper was 0, the Cobb value was 17.75 g/m^2^, and the contact angle was 95.21°, which show that the base paper had excellent hydrophobic properties, but had no oleophobic properties.

**Table 2:** Physical properties of base paper.

Basis weight (g/m^2^)	Thickness (mm)	Density (g/cm^3^)	Tensile indices (N m/g)		Kit number	Cobb (g/m^2^)	Contact angles (°)
			Longitudinal	Transverse			
110 ± 5	0.126 ± 0.004	0.87 ± 0.02	60.73 ± 0.29	34.74 ± 0.36	0	17.75 ± 0.41	95.21

Table 3 showed that the viscosity of the PVA/CNF coating was 2032 ± 37 cP, which was 103.61% higher than of the PVA coating. Therefore, the CNF increased the viscosity of coating, which was consistent with the research results of Malucelli et al.^50^. The abundant hydroxyl groups on the CNF surface, which would absorb water and swell in water, thus increasing the internal friction of the coating when it flows. In addition, the formation of stronger hydrogen bond networks between PVA and CNF hydroxyl groups was also one of the reasons why the viscosity of PVA/CNF coating was significantly higher than that of PVA coating^51^.

**Table 3:** Coating properties.

Type	pH	Apparent viscosity [cP] @ 200 rpm
PVA	6.87	998 ± 15
PVA/CNF	6.92	± 37

Note: The concentration of PVA was 10%, and the CNF dosage was 3.0% (relative to the mass of PVA).

### Grease resistance

**Figure 6:**
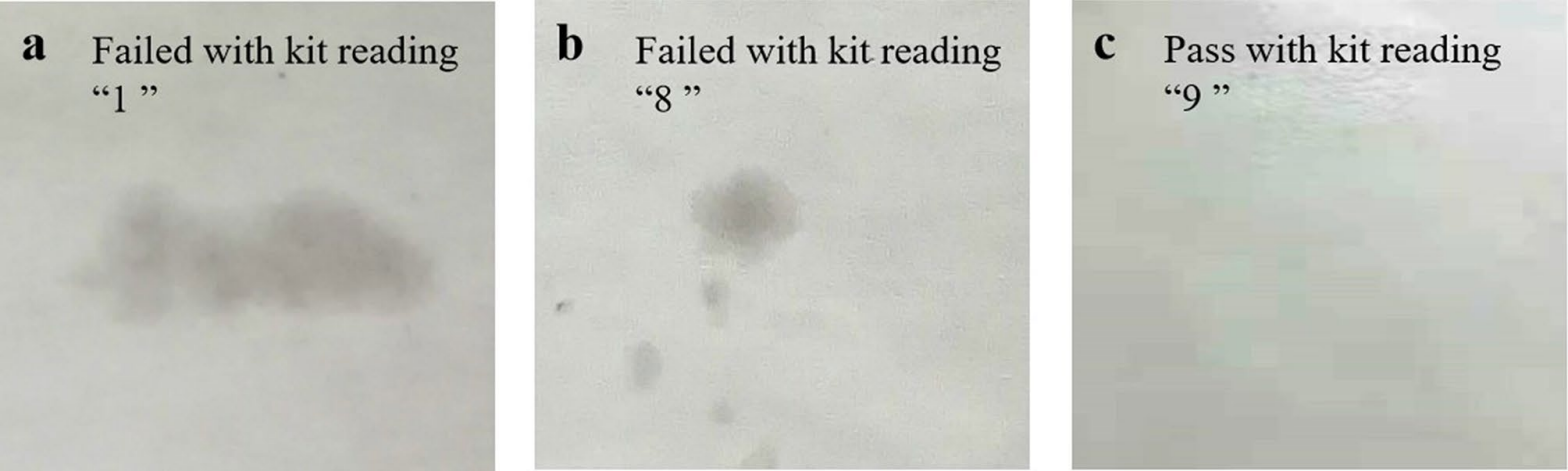
Images of kit testing of (**a**) base paper, (**b**) PVA coated paper, and (**c**) PVA/CNF coated paper.

**Figure 7:**
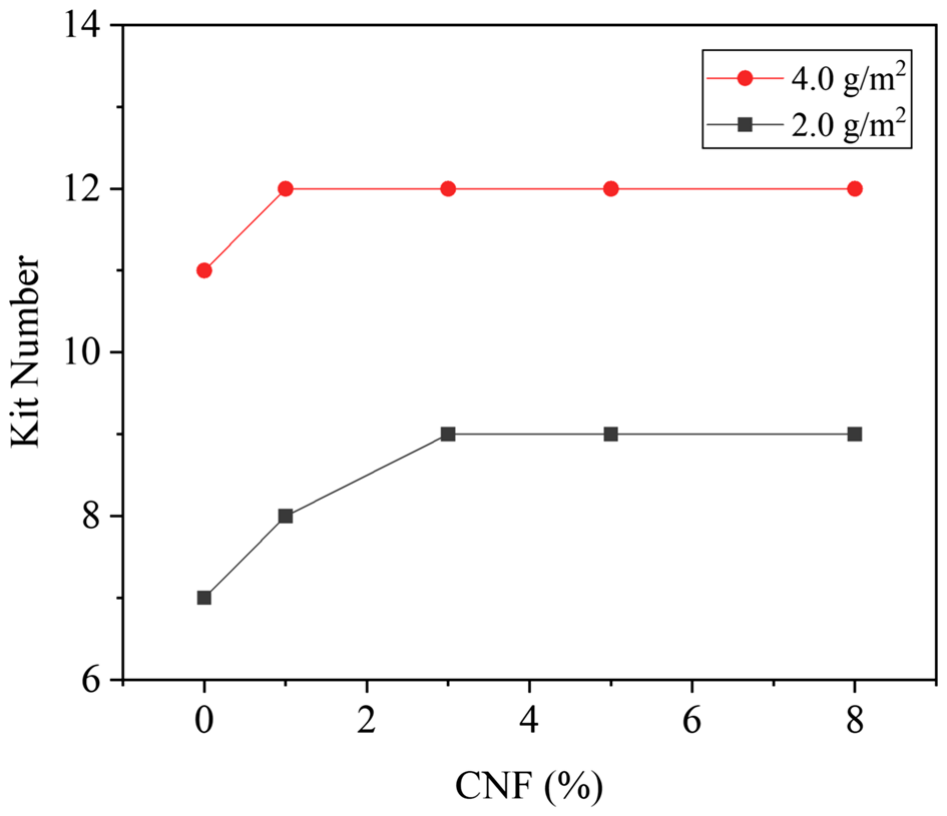
Effect of CNF dosage on grease resistance of paper.

Figure 6 shows that the grease resistance grade of base paper was 0, which does not have grease resistance performance. The effect of CNF dosage on grease resistance of paper is shown in Fig. 7. When the coating weight was 2.0 g/m^2^, the grease resistance grade of PVA coated paper was 7. The grease resistance performance was further improved with the addition of CNFs in PVA coating. When the CNF dosage was 3.0%, the grease resistance grade of the coated paper reached the highest value, which was grade 9, and then it was unchanged with the further increase of CNF dosage. When the coating weight was 4.0 g/m^2^ and the CNF dosage was 1.0%, the grease resistance grade of the PVA/CNF coated paper reached the highest level, which was grade 12.

Compared with base paper, the coated paper had better oil and grease resistance, which was due to the porous structure of base paper, that the liquid was easily absorbed and penetrated. After coating, the surface of the paper was covered with a continuous film, which greatly reduced the void size of paper, thereby reducing the penetration rate of liquids^52^. Furthermore, the addition of CNFs to the coating further improved the grease resistance of the coated paper, which was due to the CNFs increased the compactness of the coated film, preventing oil and grease from penetrating into the paper inside^11^.

### Water contact angle and Cobb value

**Figure 8:**
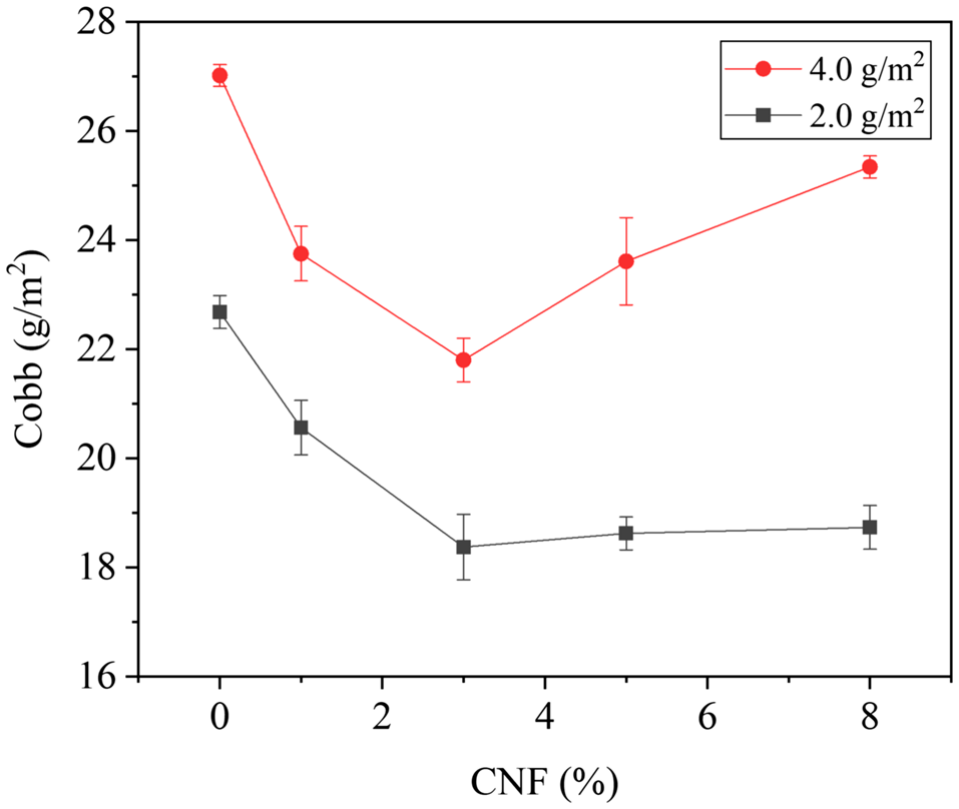
Effect of CNF dosage on hydrophobic properties of paper.

As shown in Fig. 8, with the CNF dosage increased from 0.0% to 3.0%, the Cobb value of the coated paper gradually decreased, indicating that the hydrophobicity gradually improved. When the CNF dosage exceeded 3.0%, the Cobb value began to increase, so the hydrophobicity began to decline. It may be due to that the free hydroxyl groups of PVA are completely bound to CNF, and the hydrophilic hydroxyl groups of CNFs are dominant. When the coating weight was 2.0 g/m^2^ and the CNF dosage was 3.0%, the Cobb value of coated paper reached the minimum value of 18.37 ± 0.63 g/m^2^, which was 19.00% lower than that of PVA coated paper. When the coating weight was 4.0 g/m^2^ and the CNF dosage was 3.0%, the Cobb value of coated paper was 21.80 ± 0.39 g/m^2^, which was 19.31% lower than that of PVA coated paper.

**Figure 9:**
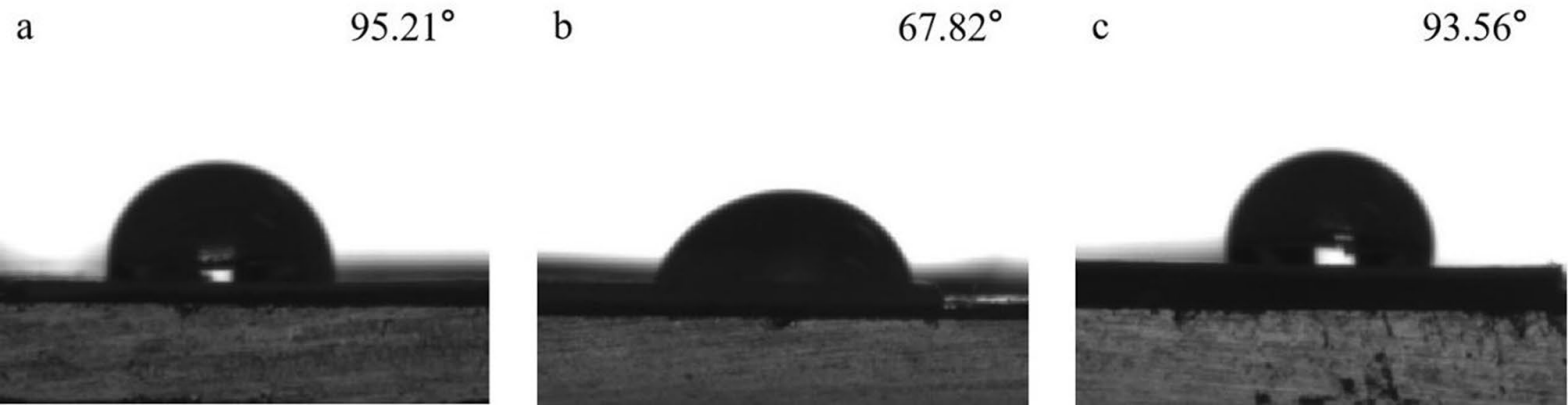
Images of contact angle test of (**a**) base paper, (**b**) PVA coated paper, and (**c**) PVA/CNF coated paper.

As can be seen from Fig. 9, the contact angle of the base paper was 95.21°, the PVA coated paper was 67.82°, and the PVA/CNF coated paper was 93.56°. Figure 9 demonstrated that the hydrophobic properties of the base paper decreased significantly after PVA coating, while the PVA/CNF coated paper basically maintained the hydrophobicity as the base paper. Due to the presence of a lot of free hydroxyl groups in PVA, the hydrophilicity of the paper coating surface is enhanced. However, with the addition of CNFs, the network structure formed between CNFs and CNFs and the hydrogen bonds between CNFs and PVA reduced the free hydroxyl groups on the coating surface, resulting in a better hydrophobic performance of the PVA/CNF coating^53^.

### Strength properties

**Figure 10:**
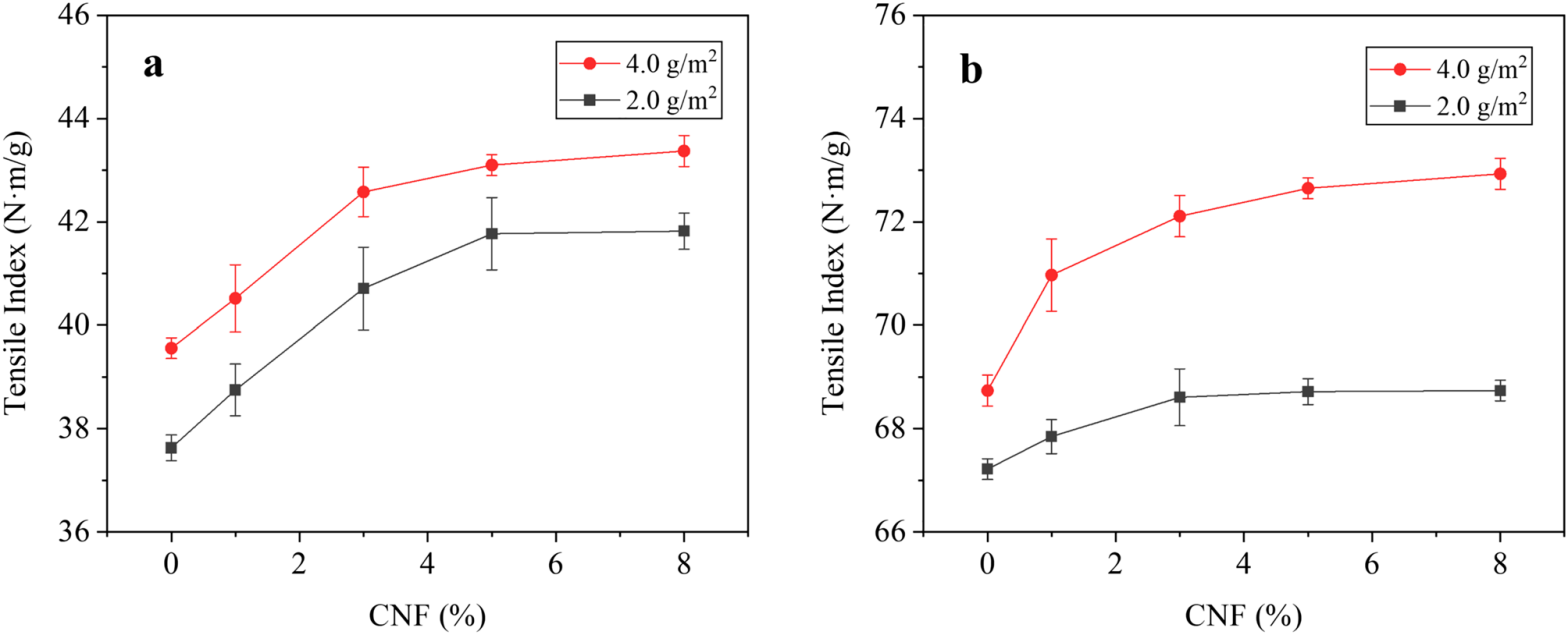
Effect of CNF dosage on (**a**) transverse and (**b**) longitudinal tensile strength of paper.

Figure 10 shows the effect of CNF dosage on the tensile strength of coated paper. As shown in Fig. 10, when the CNF dosage was increased from 0.0% and 3.0%, the tensile index of the coated paper increased significantly with the CNF dosage increased. However, continuing to increase the CNF dosage, the tensile index of the coated paper increased slowly. When the coating weight was 2.0 g/m^2^ and the CNF dosage increased from 0.0 to 3.0%, the transverse tensile index of the coated paper increased from 37.63 ± 0.25 N m/g to 40.71 ± 0.78 N m/g (Fig. 10a). Furthermore, when the coating weight was 4.0 g/m^2^ and the CNF dosage increased from 0.0% to 3.0%, the transverse tensile index of coated paper increased from 39.56 ± 0.22 N m/g to 42.58 ± 0.48 N m/g. As shown in Fig. 10b, the longitudinal tensile strengths of coated papers with 2.0 g/m^2^ and 4.0 g/m^2^ were 68.61 ± 0.55 N m/g and 72.11 ± 0.43 N m/g when the CNF dosage was 3.0%.

CNFs is rich in hydroxyl groups, which can combine with the fibers on the surface of paper by hydrogen bonding, and improve the bonding force between the fibers, thus improving the tensile strength of paper. In addition, CNFs and PVA form hydrogen bonds, forming a compact film on the paper surface, further improving the tensile strength of the paper^54^.

### Morphology of papers

**Figure 11:**
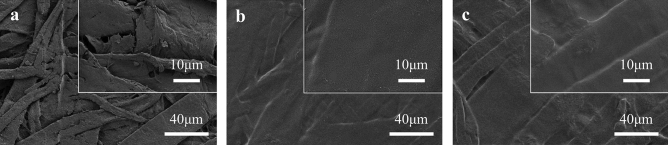
SEM images of (**a**) base paper (**b**) PVA coated paper, and (**c**) PVA/CNF coated paper.

Figure 11 shows the SEM images of base paper, PVA coated paper, and PVA/CNF coated paper. It can be observed from the Fig. 11a that the fibers of the base paper are crisscrossed, and there are a lot of gaps between the fibers. These gaps allow the liquid to penetrate the inside of the paper through capillary action, which is the main reason why the base paper does not have water and oil resistance. From the Fig. 11b,c we can observe that PVA and PVA/CNF coatings formed a smooth film on the surface of the paper, reducing the gap between fibers, thereby improving the ability of the paper to barrier oil/grease and water.

## Conclusion

This study showed that the basic chemical structures of BFs, EBFs and CNFs were similar and belong to the cellulose type I crystal. In addition, the prepared CNFs have the smallest size, with an average length of about 2.4 µm, an average diameter of about 28.7 nm, and a crystallinity of 63.63%. This study also showed that the oleophobicity, hydrophobicity, and tensile strength of the coated paper increased gradually with the increase of CNF dosage in the PVA/CNF coating solution. When the PVA/CNF coating weight was 2.0 g/m^2^ and the CNF dosage was increased from 0.0% to 3.0%, the paper grease resistance grade was increased from 7 to 9, the Cobb value was decreased from 22.68 ± 0.29 g/m^2^ to 18.37 ± 0.63 g/m^2^, the contact angle was increased from 67.82° to 93.56°, and the longitudinal and transverse tensile index were increased from 67.72 ± 0.21 N m/g and 37.63 ± 0.25 N m/g to 68.61 ± 0.55 N m/g and 40.71 ± 0.78 N m/g, respectively. Furthermore, with the increase of PVA/CNF coating amount, the hydrophobicity, oleophobicity and strength of PVA/CNF coated paper were improved. In summary, compared with the PVA coated paper, the hydrophobic and oleophobic properties of PVA/CNF coated paper are significantly improved, and the strength is maintained well. It is of great significance for the development of environmentally friendly oilproof packaging materials.

## Data Availability

All data generated or analysed during this study are included in this published article.
